# Fabrication, structural, and enhanced mechanical behavior of MgO substituted PMMA composites for dental applications

**DOI:** 10.1038/s41598-024-52202-4

**Published:** 2024-01-25

**Authors:** Savita Kumari, Rajat Kumar Mishra, Shama Parveen, Sarvesh Kumar Avinashi, Ajaz Hussain, Saurabh Kumar, Monisha Banerjee, Jitendra Rao, Rupesh Kumar, Rakesh Kumar Gautam, Chandkiram Gautam

**Affiliations:** 1 Department of Physics, University of Lucknow, Lucknow, Uttar Pradesh 226007 India; 2 Department of Zoology, University of Lucknow, Lucknow, Uttar Pradesh 226007 India; 3 Department of Prosthodontics, King George Medical University, Shah Mina Road, Chowk, Lucknow, Uttar Pradesh 226003 India; 4 Department of Mechanical Engineering, Indian Institute of Technology, Banaras Hindu University, Varanasi, UP 221005 India

**Keywords:** Biological techniques, Biophysics, Materials science

## Abstract

The most common denture material used for dentistry is poly-methyl-methacrylate (PMMA). Usually, the polymeric PMMA material has numerous biological, mechanical and cost-effective shortcomings. Hence, to resolve such types of drawbacks, attempts have been made to investigate fillers of the PMMA like alumina (Al_2_O_3_), silica (SiO_2_), zirconia (ZrO_2_) etc. For the enhancement of the PMMA properties a suitable additive is required for its orthopedic applications. Herein, the main motive of this study was to synthesize a magnesium oxide (MgO) reinforced polymer-based hybrid nano-composites by using heat cure method with superior optical, biological and mechanical characteristics. For the structural and vibrational studies of the composites, XRD and FT-IR were carried out. Herein, the percentage of crystallinity for all the fabricated composites were also calculated and found to be 14.79–30.31. Various physical and optical parameters such as density, band gap, Urbach energy, cutoff energy, cutoff wavelength, steepness parameter, electron–phonon interaction, refractive index, and optical dielectric constant were also studied and their values are found to be in the range of 1.21–1.394 g/cm^3^, 5.44–5.48 eV, 0.167–0.027 eV, 5.68 eV, 218 nm, 0.156–0.962, 4.273–0.693, 1.937–1.932, and 3.752–3.731 respectively. To evaluate the mechanical properties like compressive strength, flexural strength, and fracture toughness of the composites a Universal Testing Machine (UTM) was used and their values were 60.3 and 101 MPa, 78 and 40.3 MPa, 5.85 and 9.8 MPa-m^1/2^ respectively. Tribological tests of the composites were also carried out. In order to check the toxicity, MTT assay was also carried out for the PM0 and PM15 [(x)MgO + (100 − x) (C_5_O_2_H_8_)_n_] (x = 0 and 15) composites. This study provides a comprehensive insight into the structural, physical, optical, and biological features of the fabricated PMMA-MgO composites, highlighting the potential of the PM15 composite with its enhanced density, mechanical strength, and excellent biocompatibility for denture applications.

## Introduction

Developments in the potential uses of poly-methyl-methacrylate (PMMA) have solved numerous implementations in the area of nanotechnology. It is broadly employed in the manufacturing applications of transparent panels, replacement of glasses (for having a higher resistance to impact)^1^, as well as dentistry^2^, among others due to its good transparency^3^, stiffness, high transmittance, lightweight^4^, and supreme manufacturing ability^5^. However, there is a requirement to summarize these expansions in the terms of thoughtful and easy admittance. Herein, the flexible denture crowns are an outstanding substitute to conservatively used methyl-methacrylate dentures, which not only offer owing aesthetics and ease but also adapt to the continual movement and flexibility in partially toothless patients^6,7^. The information of the assets of PMMA has paid a lot to the current enhancements in the modification, synthesis, and uses of the polymer^2^. A PMMA's capacity to carry out in a biological system while eliciting a positive host response is biocompatibility, considered an essential biological attribute^8^. Although correctly constructed heat-cured PMMA has few biocompatibility problems, monomers remain after curing denture foundation which initiated cytotoxicity^8^, mucosal irritation, and tissue inflammation^9,10^. As a result, when mixing PMMA with more monomer solution, residual monomers are left behind, and this causes cytotoxicity ^11^. As a consequence, the research efforts have been strengthened towards to boost up the characteristics of the matrix PMMA by using the filler materials for the enhancement in the mechanical as well as for biomedical applications^12^. Additionally, PMMA has several weaknesses that bound its efficacy, like a weak radiopacity, strong exothermic reaction, etc^13^. Therefore, different kinds of fillers^14^, carbon graphite fibers, and metallic oxides are combined into the polymer matrix to overcome these complications and to boost up the desirable properties of the fabricated PMMA matrix^15,16^. Additionally, various kinds of hybrid nanofibers are achieving acceptance rapidly amongst the industries and biomaterial researchers due to their magnificent biological^17^ and mechanical properties^18,19^. The hybrid nanofibers reveal admirable properties, which attract them for several biomedical uses, such as drug delivery, wound type dressing materials, tissue engineering frames, biosensors, biological implants, and refinement devices^17^. Usually, numerous oxide-based nanostructures have attained significant focus in the research area of chemistry^20^, physics and material science^21^, because of the existence of highly electronegative oxygen atoms^20^. Bonding electrons are pulled towards itself by the higher electronegative element and isolated from the further elements accordingly, inducing substantial electric field at the interatomic scale^22,23^. Therefore, metal oxides are explicitly used as biocompatibility, optical sensors, bio-imaging, catalyst supports, adsorbents, etc^15,18^. Furthermore, amongst the numerous metal oxides, the materials having large band gap such as magnesium oxide (MgO), aluminum oxide (Al_2_O_3_), calcium oxide (CaO), and hafnium oxide (HfO_2_), are widely being used for numerous technological applications^24,25^. Strong ionic bonding among anions and cations gives rise to materials characterized by smooth surfaces, high melting points, and simple crystal structures. These qualities make them well-suited for applications in various fields such as optical devices, electronics, and advanced ceramics. Notably, Al_2_O_3_ and MgO nanoparticles, when isolated within polymeric composites, exhibit a tendency to enhance bone tissues with minimal ultimate degradation reactions^24,26^. Therefore, nanostructures have probable advanced implementations in scientific areas especially for biomedical sciences and nanomedicine owing to their efficient optical, physical, chemical^27^, and structural properties^28,29^. The metal oxides are appreciated not just due to the remarkable diversity but also due to their antibacterial properties as well as brilliant durability and mechanical properties^30,31^. Ricker et al. studied the impact of nano-sized MgO on the surface, thermal, and toxicity characteristics of PMMA and it is conveyed that nanosized MgO and PMMA enhances the radiopacity as well as compact the harmful exothermic performance of the PMMA throughout solidification^13^ Amongst the metal oxides nanoparticles, MgO play significant role because of its novel applications in various areas such as adsorption, ceramics, catalysis, electronics, coatings, detection, petrochemical products, and other fields^15,32,33^. Nanoparticles of MgO are randomly dispersed into a polymer and release Mg^2+^ ions gradually to improve the bone tissue establishment^34^ and avoid unfavorable reactions which are caused by quick degradation of Mg ions^35,36^. Soluble magnesium performs many cellular processes, acting as membrane integrity, protein synthesis, cellular respiration^17^. Additionally, MgO into PMMA matrix bone cement has a respectable tendency in bone defect filling, and joint fixation applications^34^. There is a shortage in the literature on the proficiency of this material as a filler for acrylic denture-based materials and requirements of trying in various physical as well as mechanical properties to allow worldwide receiving circumstances^6^.

In our previous work, we fabricated PMMA-ZrO_2_ nano-composites via heat cure technique and tried to magnify the mechanical as well as biological properties^37^. It is noted that mechanical strength was enhanced up to a certain level while addition of the content of ZrO_2_, after that it started to deteriorate the strength. Therefore, the main purpose of the presented research work might be giving the productive consequences to improve the more mechanical and tribological characteristics of the PMMA matrix with addition of the fillers for dentistry applications. Therefore, the results were correlated with further outcomes such as XRD, FT-IR, UV–Vis, SEM followed by EDAX, particle size analyzer, three-point bending, tribology and cytotoxicity analysis.

## Experimental and characterizations of the composites

### Synthesis of the materials

**Figure 1:**
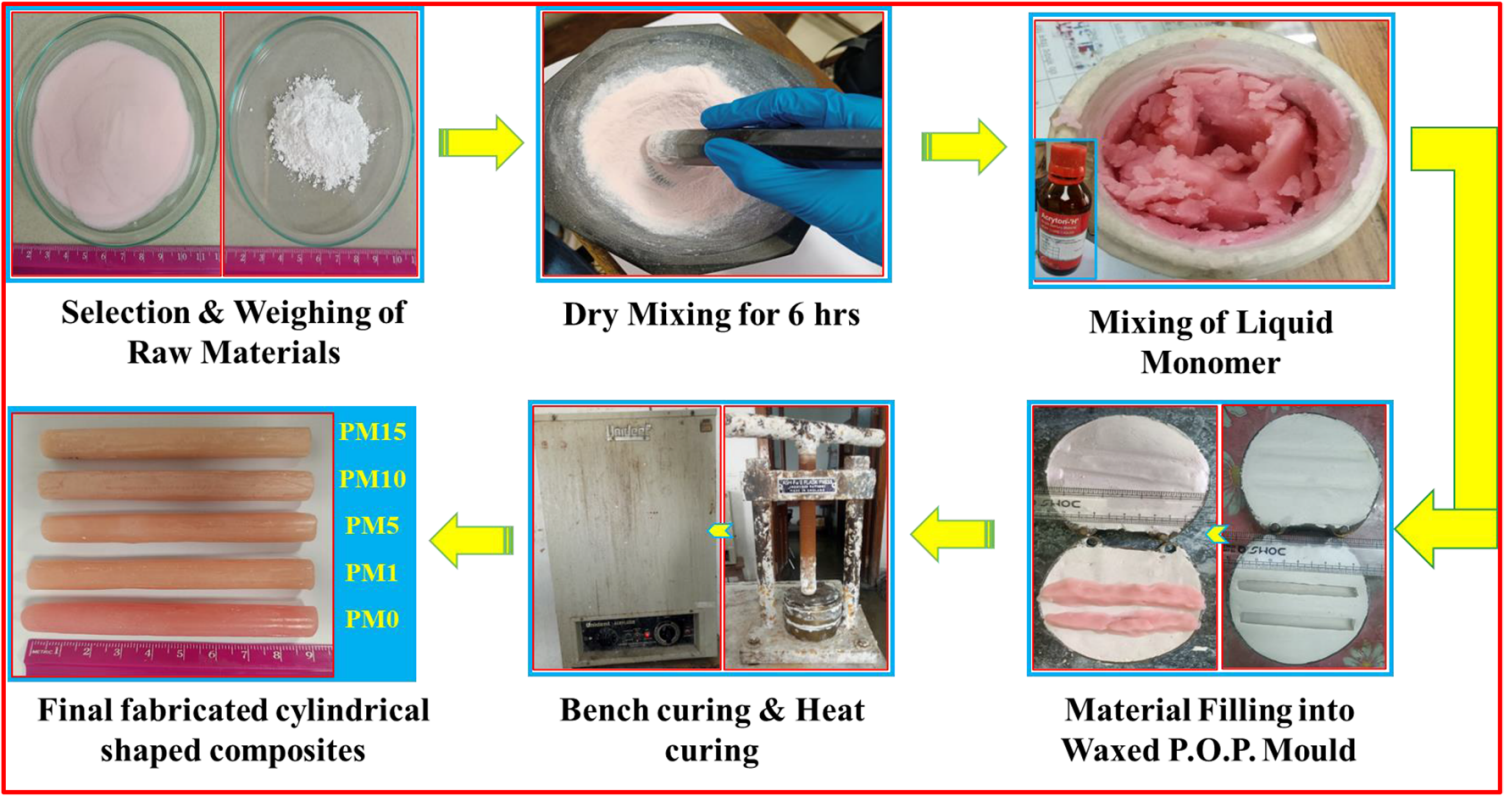
A schematic flow chart of the fabricated composites via heat cure method for a system [(x) MgO + (100 − x) (C_5_O_2_H_8_)_n_] (x = 0, 1, 5, 10 & 15 wt%) respectively.

The undoped and MgO-doped polymer-based composites were formulated via heat cure technique in the system [(x)MgO + (100 − x) (C_5_O_2_H_8_)_n_] (x = 0, 1, 5, 10 & 15 wt%). Where powdered PMMA base material (Pyrax, India), liquid (Acryton-‘H’, Orthoplast, Khurja, India) PMMA monomer, and MgO powdered form (Sigma Aldrich 99%) were previously available which are commonly used for the denture fabrications. Powdered pyrax PMMA base material already contains some amount of ZrO_2_. The hard-wax sheets of Maarc Dental (India) were cast-off to accomplish the cylindrical-shaped rods with dimensions (diameter x length; 1.3 cm x 9 cm). After the proper mixing of PMMA powder and MgO powder the liquid monomers were added into the ratio of 3:1. Synthesis of the composites and whole procedure of the technique was already discussed in previous research articles^37^. Furthermore, the procedure of the complete heat cure process is clearly shown in Fig. 1.

### XRD measurements

The structural analysis of the pure ZrO_2_, pure MgO, undoped and MgO-doped polymer composites was determined using XRD (Louisiana, USA, Rigaku Ultima-IV) containing a Cu-Kα radiation of wavelength; λ = 0.154 nm which executed at 40 kV and 40 mA. XRD patterns were recorded at the 2-theta range of 10°–70° and scanning @ 4°/min at constant room temperature (RT). The % of the crystallinity of the composites were also calculated using the Eq. (1) ^37^:

1 $$\mathrm{\%\, of\, the \,crystallinity }=\frac{\mathrm{Area \,of \,all \,crystalline \,peaks}}{\mathrm{Area \,of \,total \,peaks}}$$

### Physical parameters

Herein, several physical parameters [density (ρ), molar weight (m), and volume (v)] of the composites were calculated. The density of the cylindrical pellets of each specimen was evaluated. Density (ρ) was evaluated using the formula as^38^:

2 $$\uprho ={\text{m}}/{\text{v}}$$

where, m represents the mass of the pellet samples and v represents their volume.

### Infrared spectroscopy

IR spectroscopic study was carried out for the intermolecular interaction of the fabricated composites. For this, fine powder of mixed fabricated composites and KBr powder within a ratio of 1:100 and pressed into the form of pellets. To record the FT-IR spectra, Shimadzu made IRAffinity-1S was used in the wavenumber range of 500–4000 cm^−1^ at absolute room temperature.

### UV–Vis spectroscopy

UV–Vis spectrometer for the undoped (PM0) and MgO doped (PM1, PM5, PM10, PM15) composites was carried out using the ‘Thermo Scientific Evolution 201’ having a fixed wavelength range 200–350 nm. Further, to study the change in structures of the prepared samples their optical properties were evaluated using UV–Vis spectroscopy. Furthermore, to calculate optical energy gap (E_g_) of the samples the Tauc’s plots were plotted and briefly studied using the Eq. (3) ^37^:

3 $${\left(\alpha h\nu \right)}^{n}=(h\nu -{E}_{g})$$

where, α $\left(=\frac{4\pi k}{\lambda }\right)$ denotes absorption coefficient, hν denotes the photon energy, n = 2, represents the direct transition while E_g_ represents band gap energy of the samples.

Additionally, to check the degree of disorderness of the fabricated samples the Urbach energy (E_u_) was estimated. By using an empirical formula, the α displays the exponential nature of the photonic energy (hν) nearby the edge of absorption bands^37^:

4 $$\alpha ={\alpha }_{0} exp\left(\frac{\mathrm{h\nu }}{{{\text{E}}}_{{\text{u}}}}\right)$$

where, α_0_ a term used as constant.

Moreover, the skin depth of the fabricated composites exhibits the distance (cm) up to which an optical beam can penetrate into the composites earlier than the light beam is scattered. Hence, the Skin depth (δ) is inversely proportional to α which is formulated as^37^:

5 $$\delta =\frac{1}{\alpha }$$

The steepness parameter (S) describes the gradient of the straight line of absorption plots near the edges. The S value can be calculated by using the relation^39^:

6 $$S=\frac{kT}{{E}_{u}}$$

where, T represents the room temperature (25 ^0^C), and k denoted by Boltzmann constant (k = 1.380649 × 10^–23^ m^2^kg/s^2^K).

Furthermore, the electron–phonon interaction (E_e–ph_) parameter was calculated by using Eq. (7) which is inversely proportional to S ^39^:

7 $${E}_{e-ph}=\frac{2}{3S}$$

The refractive index (η) is the furthermost well-known optical parameter which shows the refractive characteristics of the materials with respect to the vacuum^40^.

Herein, the refractive index of the samples varies with respect to the compositions and E_g_, and calculated using below relation^40^:

8 $$\eta =\sqrt{\frac{3}{\sqrt{\frac{Eg}{20}} }-2}$$

Furthermore, the optical dielectric constant (ε) of all the undoped (PM0) and the MgO-doped fabricated samples can be evaluated using the non-linear relation^40^:

9 $$\varepsilon ={\upeta }^{2}$$

### SEM and EDAX analysis

Surface morphological study followed by EDAX of the composites were conducted by using the scanning electron microscope (JSM-6400, JEOL). Further, the smallest quantity of pellet composites were taken onto the Cu-stub and secured with a carbon tape. To make the samples conducting, a fine coating of Pt onto the pellet surfaces was employed using a Sputter coating unit (JEC-3000FC, auto fine coater JEOL). Moreover, different SEM images along with their EDAX patterns were recorded from the coated samples.

### Particle size analyzer

Particle diameter analysis of the composite samples, PM0, PM1, PM5, PM10, and PM15 were performed by the Nanozetasizer-NZS90 instrument at room temperature. It works based on the principle of dynamic light scattering (DLS) procedure and samples were organized correctly by liquefying 0.1 mg powder into the DMSO at a fixed room temperature.

### Mechanical characteristics

The mechanical behavior of the fabricated composites was performed with the help of Universal Testing Machine (UTM-Tinius Olsen H50KL) in compression mode on the pellet samples having the dimensions (diameter x length;1.3 cm × 2 cm). The Young’s modulus was also evaluated using the slope of the compressive stress (σ) and compressive strain curve^41^:

10 $$E=\frac{Stress}{Strain}$$

To analyze the load bearing performance of the undoped and doped-MgO composites, load shift curves are plotted to their constant values of compressive strain. Additionally, the fracture toughness (K_c_) of the composites was considered using area under the curve with Eq. (11) ^41^:

11 $${\text{K}}_{{\text{c}}} = {\text{ G }}\sigma \, \surd \, \left( {\pi \alpha } \right)$$

Here, G = 1, represents geometrical constant, and α is break length respectively.

Flexural strength and modulus of the undoped (PM0) and MgO doped (PM1, PM5, PM10, PM15) composites having dimensions (Diameter x length; 1.3 cm × 9 cm) were determined using three-point bending test (UTM-Tinius Olsen H50KL). The span length and the cross-head speed are 40 mm and @ 2 mm/min.

### Tribology tests

The friction and wear behavior of different MgO-reinforced PMMA composites (MgO = 0, 1, 5, 10, and 15 wt%) was investigated by performing a sliding wear test as per ASTM G99-05 standard. The test was performed using a pin of diameter `10 mm and length ~ 30 mm against a steel disc as counter material. Before any test, the sample was polished with 600 and 1500-grit emery paper to ensure uniform contact between the pin and steel disc. The weight of the sample before the test was measured by an electronic balance along with an accuracy of 0.0001 g. Further, the pin and disc surfaces were cleaned with acetone to avoid contamination. The test was carried out at 20 N and 40 N with a constant sliding speed of 0.5 m/s for a 1200 m total sliding distance. After the wear test, weight of the sample was measured by an electronic weight balance and weight loss was calculated. Then the volume loss was calculated using following formula^42^:

12 $$VL= \frac{\Delta W}{\rho }$$

where *VL* indicates the volume loss, $\Delta W$ is weight loss, and $\rho$ is the density of the composites.

The machine control unit reported the frictional force and measured the friction coefficient (*μ*).

## MTT assay

### Cell culture

SiHa, a human cervical cancer cell lines which were purchased from National center for cell science (NCCS), Pune, and grown in DMEM (Dulbecco's Modified Eagles Medium) supplemented with 2 mM L-Glutamine and 10% Fetal Bovine Serum. In an incubator, the cells were incubated at 37 °C in a 5% CO_2_ humidified atmosphere. After 70 to 80% cell confluence, the medium was removed and the cells were trypsinized for further examinations.

#### Cell viability by MTT assay

In 96-well plates, 1 × 10^4^ SiHa cells were cultivated in each well. Using the colorimetric reduction test with the tetrazolium dye 3-(4,5-dimethylthiazol-2-yl)-2,5-diphenyltetrazolium bromide (MTT) dye, the viability of both untreated and treated cells was determined^43^. The cells were exposed to various concentrations of PM0 and PM15 ranging from 50 to 250 µg/ml for 24 h before being incubated at 37 °C with 5% CO_2_. Each well received 0.5 mg/mL MTT (Himedia, Pennsylvania, United States) for three hours at 37 °C. After the medium was aspirated, 100 µl of DMSO was added to each well in order to dissolve the formazan crystals. Using a microplate reader (BioTek, Winooski, VT, USA) set to read the plate at 540 nm, the proportion of living cells was calculated. Utilising GraphPad Prism, the statistical analysis was performed. The Eq. (13) has been used to determine cell viability^43^:

13 $$\mathrm{\% Cell viability }=\frac{(\mathrm{Control \;absorbance}) - (\mathrm{test \;absorbance})}{(\mathrm{control \;absorbance})}*100$$

## Results and discussions

### XRD analysis

**Figure 2:**
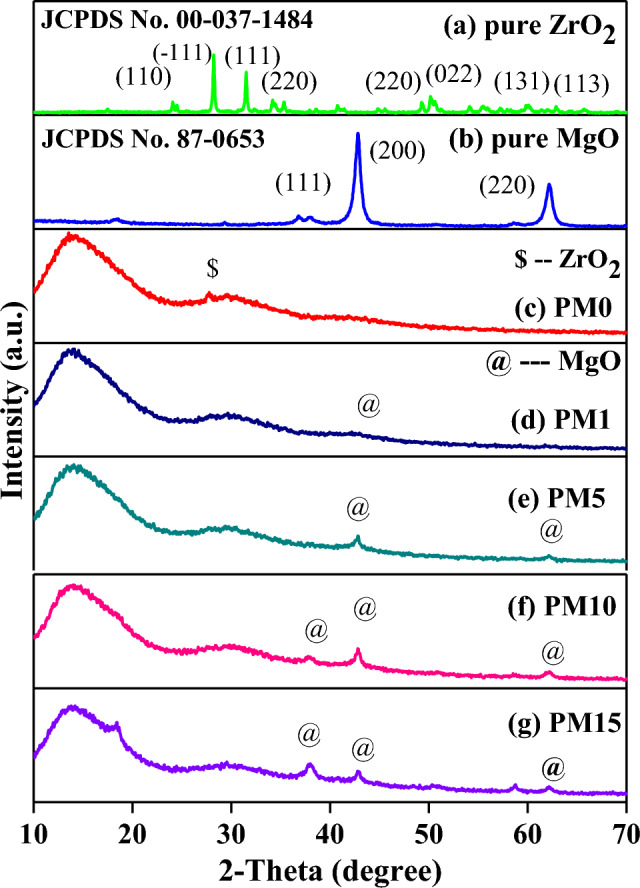
The XRD pattern of the (**a**) pure ZrO_2_, (**b**) pure MgO, and its synthesized composites; (**c**) PM0, (**d**) PM1, (**e**) PM5, (**f**) PM10, (**g**) PM15 with increasing concentration of MgO into the PMMA matrix as a system [(x) MgO + (100 − x) (C_5_O_2_H_8_)_n_] (x = 0, 1, 5, 10 & 15 wt%) respectively.

**Figure 3:**
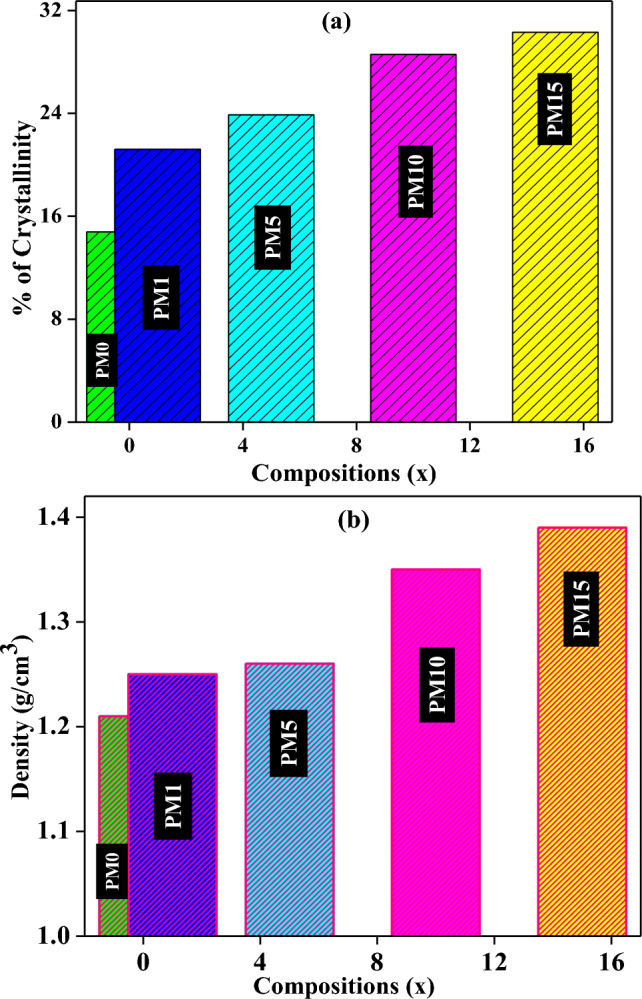
(**a**) Percentage of crystallinity and (**b**) density variations of the undoped and MgO-doped composites with varying PM (0, 1, 5, 10, 15 wt%) compositions.

The XRD analysis of pure ZrO_2_, MgO, and as fabricated composites are shown in Fig. 2a ZrO_2_, b MgO and c–g PM0, PM1, PM5, PM10 and PM15 respectively. The XRD plots confirmed that matrix has a monoclinic phase of pure ZrO_2_, and polycrystalline cubic structure of MgO particles which are recognized with their equivalent standard maximum intensity peaks and the (h k l) values are correctly marked in their successive XRD patterns. The major peaks of the XRD patterns are located at different angles, 36.8°, 42.82°, and 62.1° which corresponds to different lattice planes (111), (200), and (220) which revealed a cubic structure of MgO particles^44^. Additionally, peaks are in good agreement with their standard JCPDS File No-00-037-1484, 87-0653 of pure ZrO_2_^37^ and MgO respectively^44,45^. Figure 2c, instead of showing any sharp/crystalline peak, shows two characteristic broad humps at 2θ values of 14° and 29° of PMMA. Moreover, a low intensity peak at 27.27° corresponding to the (− 1 1 1) plane of ZrO_2_ was also noticed. Therefore, these broad humps indicate the amorphous nature of the PMMA composite^37^. However, the presence of ZrO_2_ into the Pyrax PMMA confirms the slightly crystalline nature of the PM0 composite which is already explained into our previous published research article^37^. As increasing the content of MgO into the samples, the intensity of the peak located at 42.82° is found to be increased (Fig. 2d–g). Moreover, a slight shift into this peak was observed towards the higher 2θ side. The shifting in the peaks might be attributed to a distortion in the unit cell structure of the composites^46^. Further, Fig. 3a shows the percentage of crystallinity of the fabricated composites which is determined using Eq. (1) and their values are depicted in Table 1. The percentage of crystallinity lies in the range of 14.79–30.31%. Herein, the percentage of crystallinity of the pure ZrO_2_ and MgO have been calculated as 72.28 and 61.74% respectively. Based on XRD outcomes it is concluded that the amorphousity of the composites transformed into the semi-crystalline nature in addition to the MgO content.

**Table 1:** Samples coding, various physical (ρ), particle size, % of crystallinity, optical (E_g_, E_u_, E_cut-off_, λ_cut-off_, S, E_e-ph_, η, ε) parameters, mechanical parameters; σ, strain, E, α, K_c_, flexural strength and modulus for the fabricated MgO-doped composites as a system of [(x) MgO + (100 − x) (C_5_O_2_H_8_)_n_] (x = 0, 1, 5, 10 & 15 wt%).

S. no	Samples coding →	PM0	PM1	PM5	PM10	PM15
	Parameters ↓					
1	Density (ρ) (g/cm^3^)	1.21	1.246	1.264	1.358	1.394
2	Particle Size (nm)	164	615	712	955	1110
3	% of Crystallinity	14.79	21.21	23.89	28.58	30.31
4	Direct band gap energy (E_g)_ (ev)	5.44	5.45	5.46	5.47	5.48
5	Urbach energy, E_u_ (ev)	0.167	0.098	0.047	0.036	0.027
6	Cut-off energy, E_cut-off_ (ev)	5.68	5.68	5.68	5.68	5.68
7	Cut-off wavelength, λ_cut-off_ (nm)	218	218	218	218	218
8	Steepness parameter (S)	0.156	0.265	0.553	0.722	0.962
9	Electron phonon interaction (E_e-ph_)	4.273	2.515	1.205	0.923	0.693
10	Refractive index (η)	1.937	1.936	1.934	1.933	1.932
11	Optical dielectric constant (ε)	3.752	3.747	3.742	3.736	3.731
12	Compressive strength, σ (MPa)	60.3	71.4	90.5	93.6	101
13	Compressive Strain	18.4	17.8	12.6	11.8	17.2
14	Young’s modulus, E (GPa)	0.33	0.40	0.72	0.79	0.60
15	Compressive break length, α (mm)	3.0	3.19	4.0	3.50	3.0
16	Fracture toughness, K_c_ (MPa-m^1/2^)	5.85	7.15	10.14	9.81	9.80
17	Flexural strength (MPa)	78	68.9	68.5	44.9	40.2
18	Flexural modulus (MPa)	821	732	872	563	622

### Physical parameters evaluation

The physical parameters like volume, mass and density of the fabricated composites are demonstrated in Fig. 3b and their density values are depicted in Table 1. Density of the composites is generally calculated by the atomic mass/weight, coordination numbers and atomic stack tightness. The density of the PM0 was found to be 1.21 g/cm^3^ but as increasing the MgO content into the PMMA matrix it seems to be increased with different values of 1.246 g/cm^3^, 1.264 g/cm^3^, 1.358 g/cm^3^, and 1.394 g/cm^3^ respectively. This is because the MgO is a denser material (3.58 g/cm^3^_)_ than that of the PMMA (1.20 g/cm^3^_)_, hence owing to increasing the concentration of MgO within the PMMA matrix the density of the composites gradually increased^47,48^.

### IR spectroscopy

**Figure 4:**
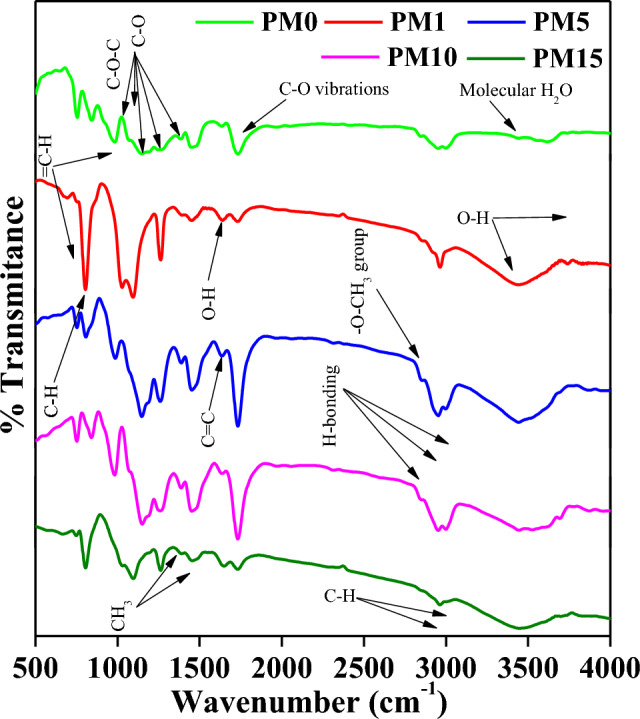
FT-IR band assignments of the undoped and MgO-doped composites with varying PM (0, 1, 5, 10, 15 wt%) compositions in transmittance mode.

The recorded FTIR spectra in transmittance mode (500–4000 cm^−1^) with their corresponding assignments of all prepared PMMA composites doped with MgO (Mole %) PM0, PM1, PM5, PM10, and PM15 are enlisted in Table 2 and demonstrated in Fig. 4. The first transmittance peak arises at 752 cm^−1^ in all samples and is attributed to the bending mode vibration of = CH group^37^. The second peak within the wavenumber range of 804–840 cm^−1^ showed the C–H vibration inside PMMA composites^49^. Further, a red shifted transmission peak at 983, 985 cm^−1^ and 981 cm^−1^ for bending mode vibration of=CH group, and also C–O–C group are assigned to these wavenumbers^37,49^. Moreover, the frequency at 1022 cm^−1^ and 1024 cm^−1^ in PM1 and PM15 are associated with the C–O stretching or O–H vibrations^50^. Next, frequency region 1064–1261 cm^−1^ was found owing to the stretching vibrations C–O molecule inside the PMMA composites^37,49^. Moreover, a peak in all samples was detected near about 1388 cm^−1^ and allotted to the bending mode vibration of –CH_2_, C=O bending vibrations, and also CH_3_ symmetric bending vibrations^37,51^. CH_3_ anti-symmetric bending vibrations were assigned for the blue shifted peaks in the wavenumber region of 1448–1452 cm^−1^^51^. The blue shifting in the transmission peak was aroused probably due to the doping content of MgO. Further, blue shifted peaks in the frequency region of 1631–1645 cm^−1^ were originated and ascribed to the C=C vibration, and stretching vibration of O–H bonds^37,52^. Next, a peak at 1730 in all PMMA composite samples was detected and assigned to the C–O vibration^52^. Furthermore, blue shifted transmission peaks in region 2846–2854 cm^−1^, assigned to the –O–CH_3_ group^53^, and H-bondings^54,55^. A stretching asymmetric –CH bond of CH_3_ group or Hydrogen bonding was attributed for the detected wavenumber region 2951–2961 cm^−1^^55,56^. Further, a transmission peak at 2997 cm^−1^ was found in PM0, PM5, PM10 due to stretching asymmetric –CH vibrations, H-bonding but this peak was found absent in PM1, PM15 samples, probably due to the incorporation of MgO^37,55^. Eventually, on increasing the MgO content the hygroscopic behaviour of samples was increased and transmission peaks in regions of 3437–3741 cm^−1^ was assigned to the –OH vibrations, and also molecular water (H_2_O)^54,55^.

**Table 2:** FT-IR band assignments of the synthesized PM (0, 1, 5, 10, & 15 wt %) composites.

S. no	Wavenumbers (cm^-1^)					FTIR band assignments	References
	PM0	PM1	PM5	PM10	PM15		
1	752	752	752	752	752	=C–H bending mode of vibrations	^37^
2	840	804	806	840	804	C–H vibration	^49^
3	983	–	985	981	–	=C–H bending mode of vibrations, C–O–C group	^37,49^
4	–	1022	–	–	1024	C–O stretching or O–H deformation	^50^
5	1070	1095	1066	1064	1097	C–O stretching vibrations	^37,49^
6	1147	–	1147	1149	–	C–O stretching vibrations	^37,49^
7	–	–	1193	1193	1195	C–O stretching vibrations	^49^
8	1249	1261	1261	1246	1261	C–O stretching vibrations	^49^
9	1384	1388	1388	1388	1388	–CH_2_ bending mode, C = O bending vibrations, CH_3_ symmetric bending	^37,51^
10	1448	1450	1450	1450	1452	CH_3_ anti-symmetric bending	^51^
11	1631	1637	1637	1637	1645	Stretching vibration of O–H bonds, C = C	^37,52^
12	1730	1730	1730	1730	1730	C–O vibration	^52^
13	2846	2852	2852	2854	2854	–O–CH_3_ group, Hydrogen bonding	^53,55^
14	2951	2962	2954	2953	2962	Stretching & asymmetric –C–H bond of CH_3_ group, Hydrogen bonding	^55,56^
15	2997	–	2997	2997	–	Asymmetric C–H stretching vibration, Hydrogen bonding	^37,55^
16	–	3437	3447	3444	3441	O–H vibration, Molecular water	^54,55^
17	–	3741	–	3695	3695	OH group	^55^

### Optical analysis

**Figure 5:**
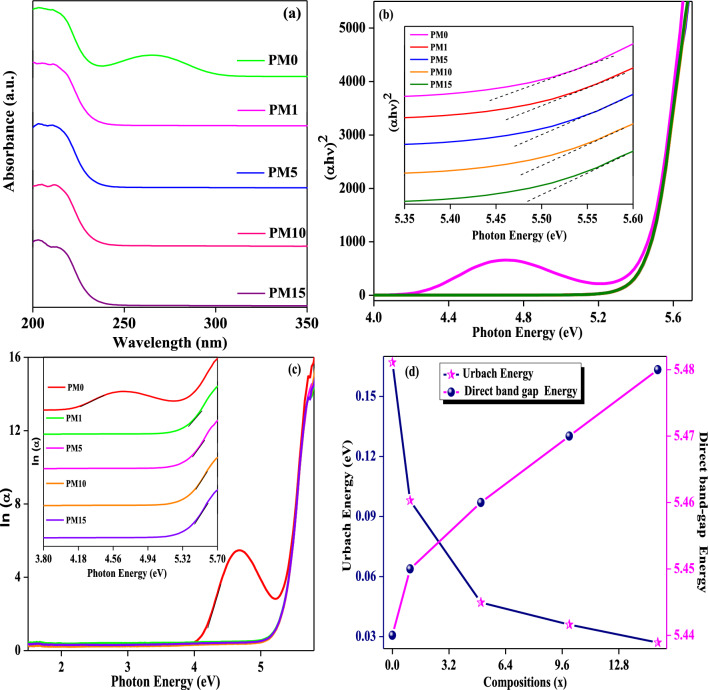
(**a**) UV–Vis absorption spectra with respect to the wavelength, (**b**) Tauc plot, (**c**) Urbach energy plot and (**d**) Cumulative graph of band gap energy and the Urbach energy with the various compositions of the synthesized composites having a system [(x) MgO + (100 − x) (C_5_O_2_H_8_)_n_] (x = 0, 1, 5, 10 & 15 wt%).

Optical properties as synthesized composites, PM0, PM1, PM5, PM10, and PM15 are quantified by employing an UV–Vis spectroscopy using Eq. (3) and revealed in Fig. 5a respectively. The absorption of light through the samples in the UV–visible region comprises the elevation of the electrons in σ, π, η orbital from ground to higher energy state^56,57^. Therefore, the PM0 exhibits a strong absorption edge produced owing to the electronic excitation within the C=O bond presented in the PMMA. The absorption band arises at 203 nm, this might be corresponding to π–π^*^ transition of carbonyl chromophores (C=O) bonds (Fig. 5a)^58^. Another band was also observed within the wavelength range of 250–300 nm which might be owing to an increased number of PMMA molecules. Moreover, these peaks are ascribed to the forbidden n–π* transition that is less intense in comparison to the π–π* transition. However, in doped MgO composites both of the bands show broadening along with saturation in the absorption^58^, and shows unstructured behavior of the composites as well as the absorption edge is showing a red shift (moving towards higher wavelength) which confirms the semicrystalline nature of the composites. Therefore, the nonappearance of the sharp peaks claims about the small bandgap of fabricated composites. Figure 5b exhibits the Tauc plots where (αhν)^2^ varies with the function of the photon energy (hν). Herein, the direct band gap energy of the un-doped sample (PM0), and doped MgO samples, PM1, PM5, PM10, and PM15 was calculated using Eq. (3) and their values are 5.44, 5.45, 5.46, 5.47, and 5.48 eV which reveals the insulating behavior of the composites (Table 1). Moreover, the slight difference in band gap energy between the optical bands suggested a potential for the composites to facilitate ion transfer between PMMA and MgO particles ^59^. Furthermore, Urbach energy is also quantified by using Eq. (4), which tells about the degree of randomness into the fabricated composites. In Fig. 5(c), Urbach energy of the un-doped MgO (PM0) composite was observed to be 0.167 eV which reveals the amorphous nature of the material^37^. As increasing the MgO into the PMMA matrix the Urbach energy starts to decrease from 0.098 to 0.027 eV respectively (Table 1). Therefore, increasing MgO into the PMMA system decreases the values of Urbach energy which further reveals a decrease in the disorderness into the matrix^60^. Figure 5d exhibits a cumulative variation of Urbach energy and the direct band gap energy with the varying concentrations of the MgO into the PMMA matrix.

**Figure 6:**
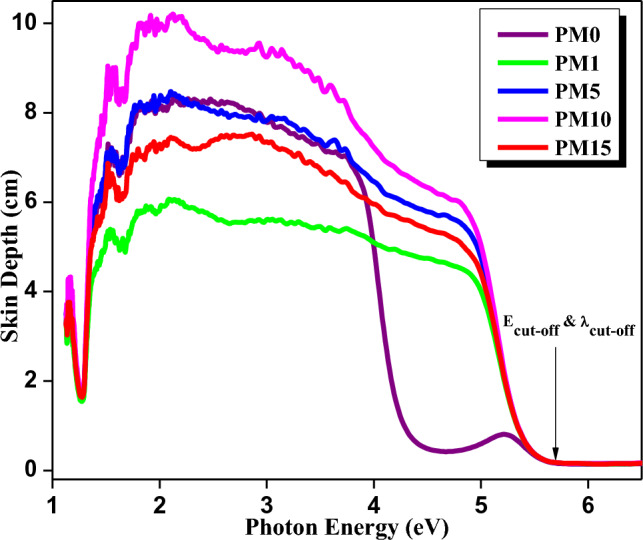
The skin depth variations with photon energy for the fabricated composites.

Furthermore, the values of the skin depth, δ (cm) was calculated using Eq. (6) and the graph was plotted (δ vs. hυ) as shown in Fig. 6. Therefore, the values of cut-off wavelength (λ_cut-off_) correspondingly cut-off energy (E_cut-off_) were found to be 218 nm and 5.68 eV for all the fabricated composites respectively (Table 1).

**Figure 7:**
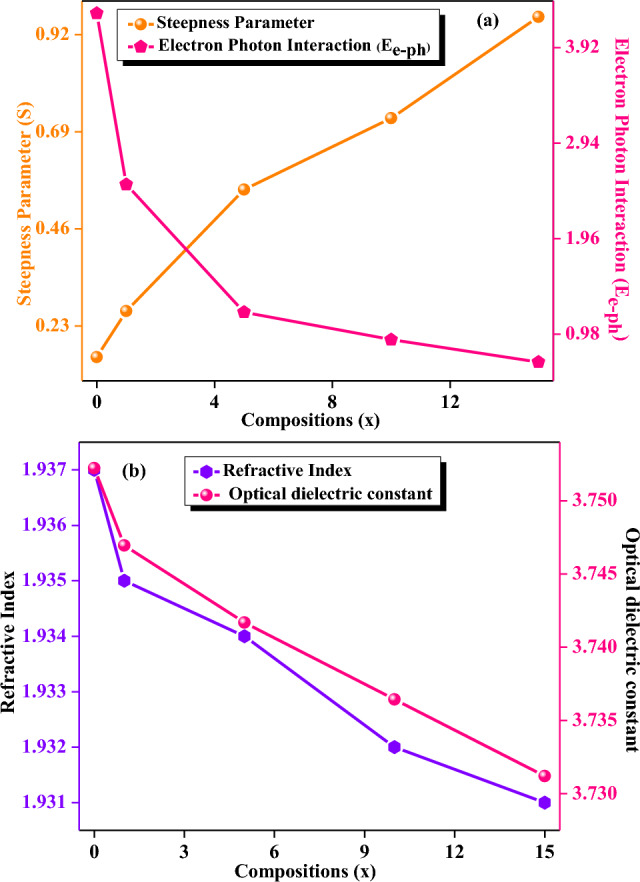
Variations of (**a**) Steepness parameter and electron phonon interaction (**b**) refractive index and optical dielectric constant with respect to the varying compositions in the system of [(x) MgO + (100 − x) (C_5_O_2_H_8_)_n_] (x = 0, 1, 5, 10 & 15 wt%).

The steepness parameter (S) values of the undoped (PM0) and MgO doped (PM1, PM5, PM10, and PM15) composites are calculated using Eq. (6). Herein, S is inversely proportional to E_u_. The increasing behavior of S values might be due to decrement in E_u_ values and enlisted in Table 1. Additionally, E_e-ph_ interaction for all fabricated composites were evaluated using Eq. (7) and values are listed in Table 1. As increasing the dopant MgO into the PMMA matrix, the E_e-ph_ interaction starts to decrease from the 4.273, 2.515, 1.205, 0.923, and 0.693 respectively. This might be happening due to difference in present anion charges as well as iconic effect in the matrix^39^. Therefore, Fig. 7a shows the increment and decrement behavior of S and E_e-ph_ interaction of the fabricated composites as increasing the MgO dopant into the matrix.

The refractive index (η) was also calculated using Eq. (8) and the obtained values are depicted in Table 1. As the dopant concentrations increased, the value of η gradually decreases from 1.937 to 1.932 (Fig. 7b). Moreover, optical dielectric constant (ε) was also evaluated using Eq. (9) and the values of ε decreases as increasing the concentration of MgO into the PMMA matrix such as 3.752, 3.747, 3.742, 3.736, and 3.731 respectively (Table 1). The values of ε for synthesized composites decreased owing to the non-linear relation with η. Therefore, Fig. 7b exhibits a relationship between the refractive index (η) and the ε with the varying concentrations of the MgO into the PMMA matrix. Both values are showing a decreasing behaviour with the compositions (x) because both are inversely proportional to the band gap energies of the composites.

### Morphological analysis

**Figure 8:**
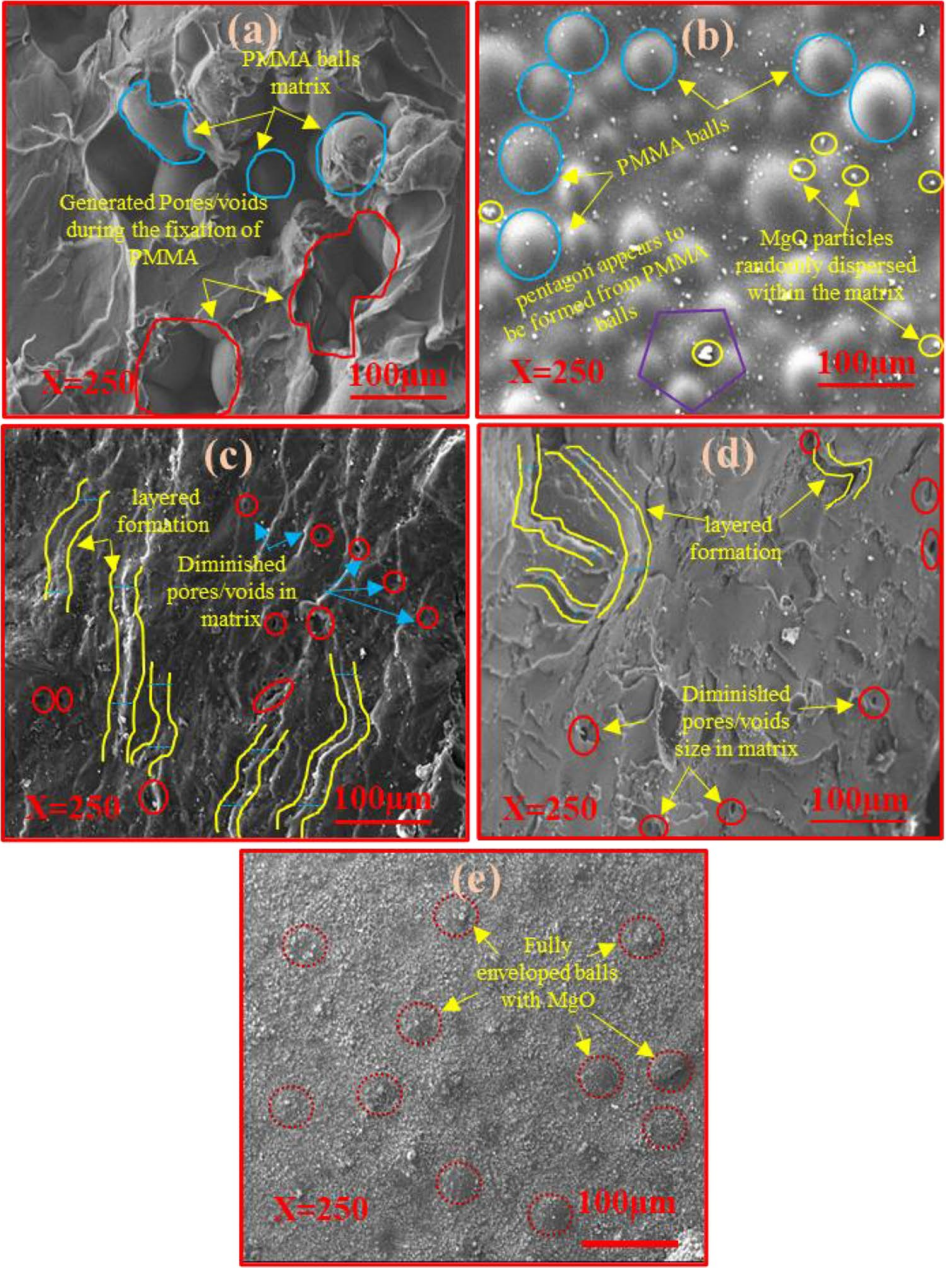
(**a**–**e**) Scanning electron micrographs of the undoped and MgO doped polymer based fabricated (PM0, PM1, PM5, PM10, PM15) composites at magnification; X = 250 respectively.

SEM micrographs of all the fabricated composites at a fixed magnification of × 250 are shown in Fig. 8a–e. Figure 8a shows a matrix which is fully shielded with PMMA balls and also some of the generated pores/voids were noticed which might be created during the fixation of the PMMA. These voids affect the mechanical properties significantly of the fabricated composites. So, to overcome such imperfection, the MgO content was added into the PMMA matrix which might be useful to enhance the mechanical stability^48^. The addition of 1wt% of MgO into the PMMA caused the MgO powder to be randomly dispersed and distributed all over the composite (PM1). However, the PMMA balls were not fully wrapped with MgO particles which indicates that there is no strong intermolecular association in-between PMMA and the MgO particles as depicted in Fig. 8b ^33^. Further, as the MgO content increased from 1 to 5%, randomly distributed MgO particles are clearly seen to be decreasing and started merging with the PMMA matrix which indicates better interaction within the fabricated composites (Fig. 8c). The layered PMMA structure has also been observed which is partially filled with the MgO particles, which might be owing to the newly deformation or formation of various types of bonding which are also observed in their FTIR spectrums. Additionally, the diminished pores and voids were observed within the composite PM5. The PMMA layered structure has been further filled with the MgO at increasing the content of MgO by 10 wt% (i.e., PM10) into the PM0 matrix and size of the voids were found to be reduced (Fig. 8d). Further, in addition to 15 wt% of MgO, all voids have been completely filled with the MgO particles and not a single void was seen in the fabricated PMMA matrix of the composite PM15 in Fig. 8e. Additionally, the PMMA balls have been fully enveloped/wrapped with the MgO particles which might be helpful to boost up the mechanical characteristics of the synthesized composites. But the phase separations in the micrographs were also observed. They might be owing to the lack of homogeneity between the PMMA and previously existing ZrO_2_ so these shortcomings may affect the flexural properties of the fabricated composites^61^.

### Elemental analysis

**Figure 9:**
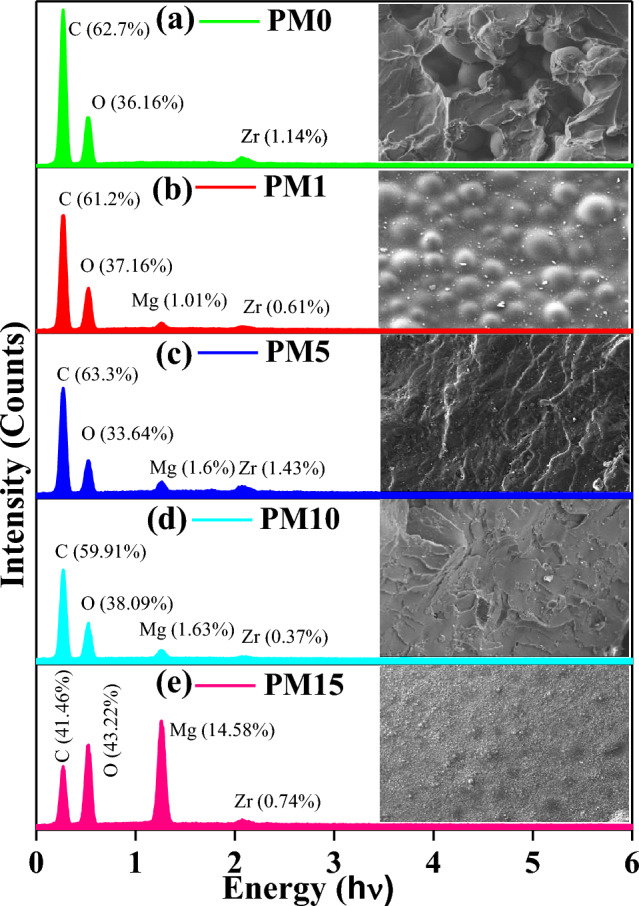
Energy dispersive X-ray analysis (EDAX) spectrums of undoped (**a**) PM0, and MgO-doped (**b**) PM1, (**c**) PM5, (**d**) PM10, (**e**) PM15 fabricated composites for a system [(x) MgO + (100 − x) (C_5_O_2_H_8_)_n_] (x = 0, 1, 5, 10 & 15 wt%) respectively.

The elemental analysis of the undoped and MgO-doped fabricated composites is shown in Fig. 9a–e, and the values of their compositions (weight % and atomic %) are listed in Table 3. Herein, Fig. 9a displays the average distribution of distinct elements onto the surface of the matrix, where 62.7% of carbon (C), 36.16% of oxygen (O), and 1.14% of zirconia (Zr) respectively. Here, Zr is already presented into the Pyrax PMMA material which is matched with the previously XRD results and the presence of O concludes the contribution of the oxides into the fabrication of the samples. As increasing the MgO into the previous matrix, the intensity of the peaks varies such as C (61.22%), O (37.16%), Zr (0.61%), and having a new peak of Mg (1.01%) that was also observed into the EDAX spectrum (Fig. 9b). Therefore, PMMA and the MgO particles have no strong intermolecular bonding which is already discussed in the SEM results. Further, on adding the MgO contents of 5 and 10 wt% into the PMMA matrix, the layers, and pores formation was observed in both micrographs and the elemental compositions were seems to be randomly varying of C, O, Mg, and Zr (63.33%, 33.64%, 1.6%, 1.43% and 59.91%, 38.09%, 1.63%, 0.37%) respectively. Furthermore, the EDAX spectrums of all the synthesized composites exposed an average elemental composition of C, O, Mg and Zr without any kind of impurities. Herein, on adding the 15 wt% of MgO, the Mg has the highest intensity peak and has its wt% content of 14.58%. Therefore, the balls of PMMA have been entirely wrapped with the MgO particles which might be enhancing the optical as well as mechanical performances of the fabricated composites. Herein, the atomic % is varying similarly with the wt% of the fabricated composites.

**Table 3:** Samples coding, elements, weight %, and atomic % of the synthesized PM (0, 1, 5, 10, & 15 wt %) composites.

Samples coding	Elements	Weight %	Atomic %
PM0 Fig. (a)	C	62.7	69.67
	O	36.16	30.16
	Zr	1.14	0.17
PM1 Fig. (b)	C	61.22	68.25
	O	37.16	31.1
	Mg	1.01	0.56
	Zr	0.61	0.09
PM5 Fig. (c)	C	63.33	70.71
	O	33.64	28.19
	Mg	1.6	0.88
	Zr	1.43	0.21
PM10 Fig. (d)	C	59.91	67.04
	O	38.09	32
	Mg	1.63	0.9
	Zr	0.37	0.05
PM15 Fig. (e)	C	41.46	51.06
	O	43.22	39.95
	Mg	14.58	8.87
	Zr	0.74	0.12

### Particle size analyzer (PSA) study

**Figure 10:**
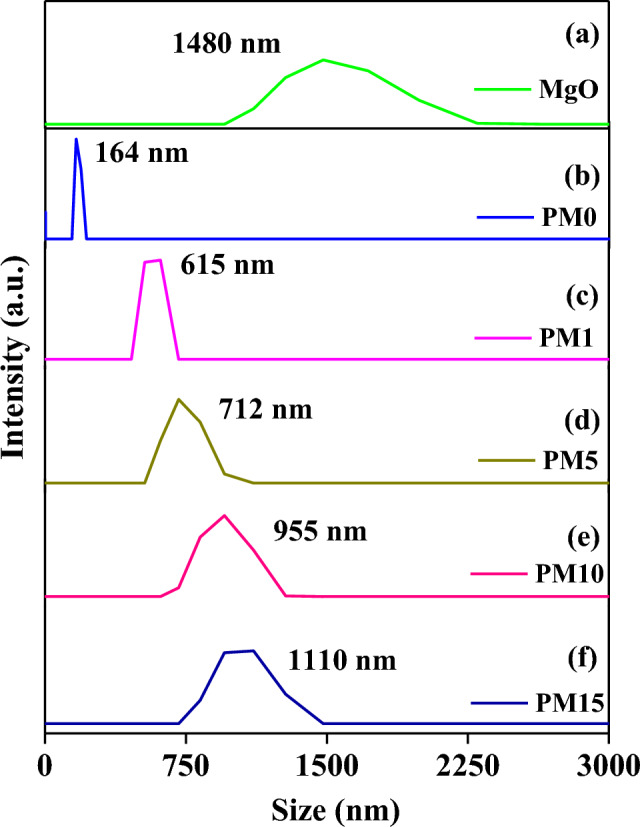
Particle size distribution of (**a**) pure MgO, and synthesized undoped and MgO-doped resin-based composites (**b**) PM0, (**c**) PM1, (**d**) PM5, (**e**) PM10, (**f**) PM15 respectively.

The particle size diameter (PSD) of pure MgO and as prepared composites, PM0, PM1, PM5, PM10, and PM15 are enlisted in Table 1 and its graph is shown in Fig. 10a–f respectively. Herein, the PSD of the PM0 is found to be 164 nm, but as increasing the MgO into the previous matrix the PSD values starts to be increasing such as 615 nm, 712 nm, 955 nm, and 1110 nm respectively. This might be happening due to the larger PSD value of the pure MgO than the PMMA matrix, which is observed to be 1480 nm^62^. The variations into the PSD will be helpful for the further mechanical properties but size of the particle does not play any important role into the flexural strength of the composites^62,63^.

### Mechanical behavior

**Figure 11:**
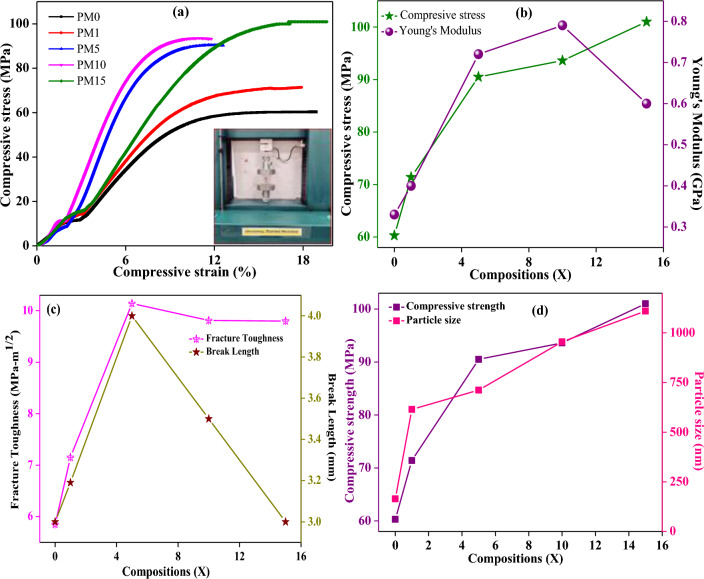
Mechanical behavior of the fabricated composites with (**a**) variations of compressive stress vs strain (inset showing the test in compression mode), (**b**) compressive stress and Young’s modulus, (**c**) fracture toughness and break length, (**d**) compressive strength and particle size with varying compositions of MgO for a system of [(x) MgO + (100 − x) (C_5_O_2_H_8_)_n_] (x = 0, 1, 5, 10 & 15 wt%).

**Figure 12:**
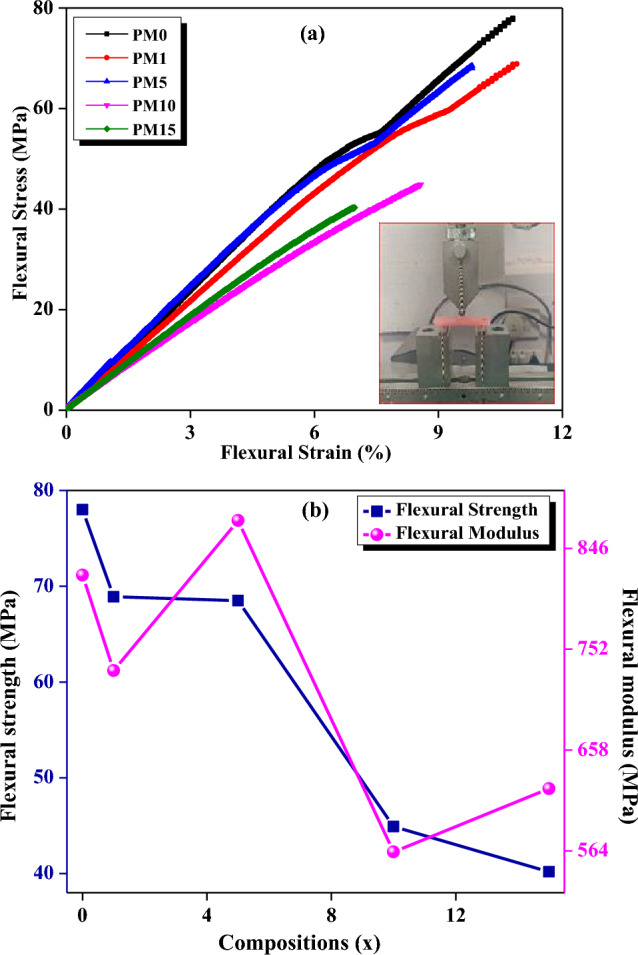
(**a**) Variations of flexural stress vs strain (inset display the 3-point bending test), (**b**) plot between the flexural strength and flexural modulus with increasing (0, 1, 5, 10, 15) wt% of MgO into the resin matrix using a three-point bending test.

The mechanical properties such as compressive strain, stress, fracture toughness, Young’s modulus, break-length etc. of the undoped (PM0) and MgO-doped (PM1, PM5, PM10, and PM15) composites are shown in Fig. 11a–c and their values are recorded in Table 1. The stress increases while increasing the strain and approaches up to a plateau and attends to their constant values (Fig. 11a). The compressive stress of undoped (PM0) composite is obtained to be 60.3 MPa. Further, increasing the dopant concentration of the MgO into the PMMA matrix the strength of the composite tends to be increasing from 71.4 to 101 MPa. So, PM15 indicates the increased rigidity of the fabricated composite^64^. This happens due to the PMMA balls having been totally enveloped with the increasing MgO particles which is clearly observed and matched with the SEM results. Therefore, it concludes that MgO filler enhances the mechanical strength of the PMMA composite up to 68%. Further, Young’s modulus was also evaluated by via the stress–strain curve (Eq. 10) and values are found to be 0.33 GPa, 0.4 GPa, 0.72 GPa, 0.79 GPa, and 0.60 GPa for all the undoped and doped-MgO composites respectively (Table 1). Moreover, in Fig. 11b, the compressive strength and Young’s modulus of the composites were seeming to increase while adding dopant MgO in 1, 5, 10, and 15 wt% into the PM0 matrix. Furthermore, the fracture toughness of the synthesized composites was also determined using Eq. (11) and obtained from 5.85 to 9.80 MPa-m^1/2^ for PM (0, 1, 5, 10 and 15 wt%) respectively Table 1). Herein, enhancing the fracture toughness of the composites might be owing to the crack’s deflection, particles bridging at the extremes of matrix/particle interface^65^, which is clearly visible in the SEM micrographs (Fig. 8). The fracture toughness and break length Vs various compositions of un-doped and doped MgO were also determined and revealed in Fig. 11c. However, it may be owing to the filled pores by the MgO over the PMMA matrix. Thereafter, a graph was plotted between the strength and the particle size Vs varying compositions of MgO and observed that as increasing the MgO into the matrix the strength and the particle size of the composites increased rapidly (Fig. 11d). Herein, larger particle size of the MgO particles is responsible for enhancing the particle size of the doped composites. These results are also correlated with the previous XRD and SEM micrographs. Flexural strength and modulus of the undoped and MgO doped resin-based composites were determined using the three-point bending test which is clearly shown in Fig. 12a and values are depicted in Table 1. Figure 12a, shows the variations between the compressive stress and strain of the composites. Herein, as increasing the MgO dopant (0, 1, 5, 10 and 15 wt%) the flexural stress of the samples starts to be decreasing from 78 to 40.2 MPa. Figure 12b, exhibits the variations of flexural strength and the flexural modulus with fabricated composites. Flexural modulus of the undoped (PM0) composite was obtained to be 821 MPa, but increasing MgO doping content from 1 to 15% the values of modulus degrades from 732 to 622 MPa as well as the flexural strength respectively (Table 1). Therefore, inhomogeneity or poor adhesion between the polymer material and the metal oxides might be the one of the reasons for the decreasing flexural properties^61,65–67^.

### Tribology analysis of the composites

**Figure 13:**
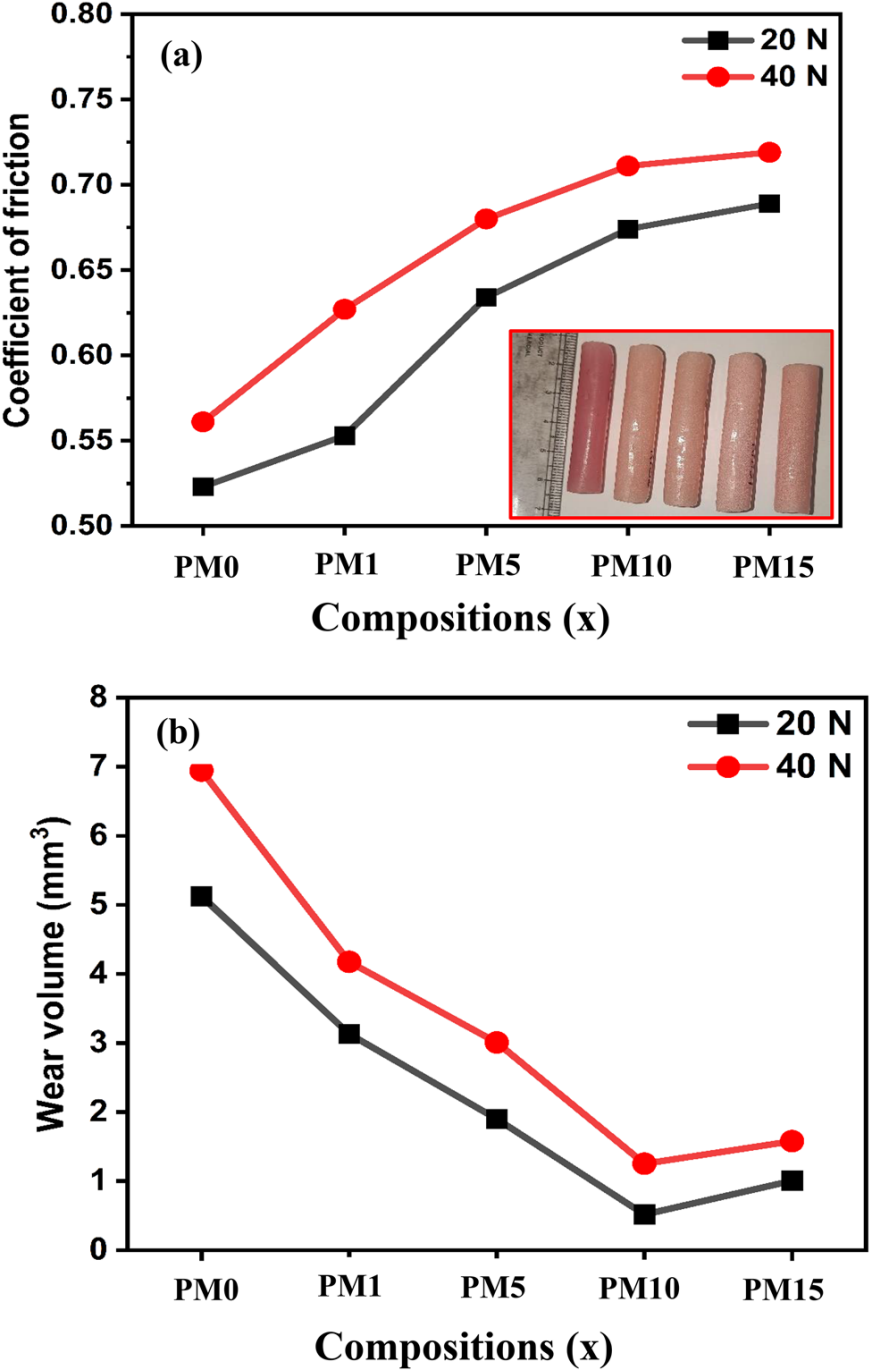
(**a**) Coefficient of friction (inset showing the samples dimension for tribology test), and (**b**) Wear volume of different MgO reinforced composites at 20 and 40 N normal load.

Figure 13a shows the coefficient of friction (COF) of MgO added PMMA composites at 20 N and 40 N, respectively. The PMMA without reinforcement (PM0) shows COF of 0.523 at 20 N and 0.561 at 40 N normal load. Adding MgO up to 10% significantly increases the COF value. Above that, the increment in COF is nominal, as seen in Fig. 13a. This increase in COF is mainly contributed by adding hard MgO particles to the soft PMMA matrix. MgO particles get stuck in the asperities and cause scratch effects resulting in an increase in the values of COF for the MgO reinforced composites^68^. It is also observed that the slope of increment in COF decreases with the concentration of MgO. A similar behavior of MgO reinforcement is also observed for the 40 N load. However, the COF at 40 N is considerably higher compared to 20 N and the highest value of COF obtained for PM15 samples are 0.689 and 0.719 at 20 N and 40 N, respectively. The wear volume of MgO-reinforced PMMA composites is shown in Fig. 13b. The wear volume obtained for pure PMMA (PM0) is notably higher than the PMMA reinforced with MgO. It is observed that the MgO reinforcement has improved the antiwear behavior of PMMA composites. The volume loss reduces significantly with the reinforcement of MgO up to 10 wt% and then increases slightly at 15 wt% which can be noticed in Fig. 13b. The highest and lowest wear volume for this composite is obtained as 5.12 mm^3^ and 0.52 mm^3^ at 20 N load. Similarly, the highest and lowest volume loss obtained at 40 N load is 6.94 and 1.25 mm^3^, respectively. Additionally, the normal load also has a significant effect on the wear volume. The volume loss is remarkably higher for all composites at 40 N as compared to 20 N load, which can be observed from Fig. 13b. The high wear volume of the pure PMMA (PM0) can be correlated with high heat generation as the friction pairs result in more volume loss. However, the incorporation of MgO helps to reduce the volume loss of PMMA composites. Such improvement in the antiwear behavior can be correlated with the high-temperature stability of MgO particles due to its higher melting point (3125 K)^69,70^. Therefore, adding MgO particles into the PMMA reduces the direct contact of PMMA with the counter steel disc and reduces the wear volume of the composites. Moreover, the increment of volume loss after 10 wt% mainly happens due to the third body abrasion effect by accumulating more particles.

## MTT assay

### Cytotoxic against cervical cancer cells

**Figure 14:**
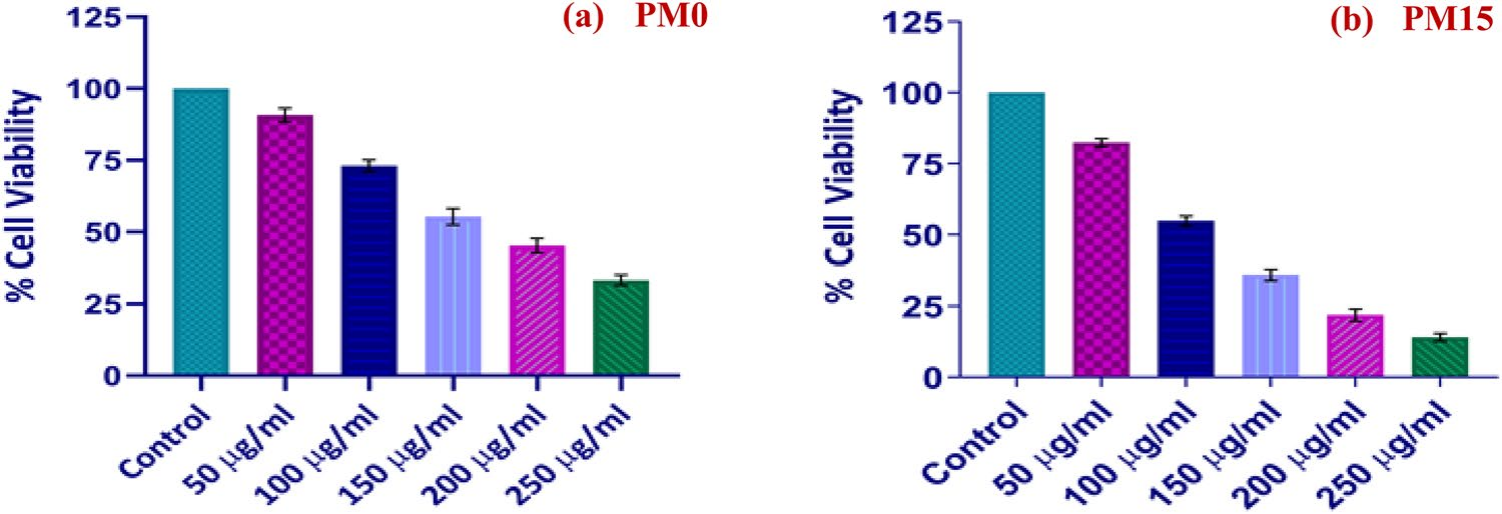
(**a** and **b**) Percent cell viability of SiHa cells treated with different concentrations of PM0 and PM15 respectively.

To check out the biocompatibility of all the synthesized composites, PM0, PM1, PM5, PM10, and PM15, the MTT assay was performed for the lowest and highest content of MgO dopant: PM0 and PM15 composite sample. The highlights of the data were clearly drawn in Fig. 14a, b for both composites. The viability of (SiHa) cells is used to determine the cytotoxicity of the compounds. As we substitute the PM0 synthesized material with the control group with different concentrations from 50 to 250 µg/ml, a decaying division of the formazan was apparent with respect to the (untreated cells) control group cultures (Fig. 14a). Therefore, these results clearly demonstrated that, relative to control cells (untreated cells), the cell viability of cancer cells decreased significantly as the dose concentration is to be increased. Similarly, the viability of cancer cells seems to be decreased meaningfully as the dose concentrations of the (PM15) composite sample is to be increased (Fig. 14b). The half maximal inhibitory concentrations (IC50) of PM0 and PM15 were calculated ~ 160 μg/ml and ~ 120 μg/ml, respectively. This concentration is required for 50% in vitro inhibition. Therefore, both of the composites have the positive response towards the biocompatibility test but PM15 is more effective material because it has the 50% cancer cells that were dead at only ~ 120 μg/ml concentration. Furthermore, there is not any type of allergic output to contact with the oral tissue and the assay significance in the remaining study acknowledges that the above-mentioned samples have the good cytotoxicity and are utilized to fabricate the dentures.

## Conclusions

A series of composites (PM0–PM15) with system [(x)MgO + (100 − x)(C_5_O_2_H_8_)_n_] (x = 0 ≤ x ≤ 15 wt%) were synthesized via heat cure technique successfully. The amorphous nature of PMMA was evident in the XRD patterns, and as MgO content increased, a shift in peak intensity and position suggested structural variations in the composites. The density of the composites increased from 1.21 to 1.394 g/cm^3^ with increasing MgO content due to the higher density of MgO in comparison to PMMA. FTIR spectra revealed characteristic vibrations, while UV–Vis spectroscopy indicated a red shift in absorption edges, signifying the semi-crystalline nature of the composites. Morphological analysis via SEM showed effective dispersion of MgO within the matrix, influencing mechanical properties significantly. The PM15 composite exhibited the highest compressive strength (101 MPa) among all the fabricated composites. Elemental analysis, PSD studies, tribological analysis, and MTT assays demonstrated the tailored properties of the composites with improved biocompatibility. This study provides a comprehensive understanding of the structural, physical, optical, and biological characteristics of the synthesized PMMA-MgO composites. Eventually, the fabricated PM15 composite with high density, good mechanical strength, and excellent biocompatible can be used for the denture applications.

## Data Availability

Data will be provided on request from the corresponding author.
